# Assessing the Genetic Health and Conservation Value of an Introduced Urban Population of a Critically Endangered Parrot

**DOI:** 10.1111/eva.70245

**Published:** 2026-05-14

**Authors:** Astrid A. Andersson, Arthur F. Sands, Kerry Reid, Taylor Hains, Jessica Lee, Frank E. Rheindt, Juha Merilä, Caroline Dingle

**Affiliations:** ^1^ Area of Ecology and Biodiversity, School of Biological Sciences University of Hong Kong Hong Kong SAR; ^2^ Avian Evolution Laboratory, Department of Biological Sciences National University of Singapore Singapore; ^3^ Committee on Evolutionary Biology University of Chicago Chicago Illinois USA; ^4^ Negaunee Integrative Research Center The Field Museum Chicago Illinois USA; ^5^ Mandai Nature Singapore; ^6^ Ecological Genetics Research Unit, Organismal and Evolutionary Biology Programme University of Helsinki Helsinki Finland; ^7^ Capilano University North Vancouver British Columbia Canada

**Keywords:** admixture, Cacatua, conservation genetics, genetic diversity, Hong Kong, inbreeding

## Abstract

Non‐native species can be introduced to novel environments such as cities via wildlife trade. These populations may have conservation value—particularly if they are of a species threatened in its native range. Genetic tools can help assess the fitness of introduced populations by indicating if they are (1) suffering the consequences typically associated with small, isolated populations derived from few founders (e.g., inbreeding, low genetic diversity) or (2) they demonstrate gene flow indicative of mixed‐origin founders, successful breeding, and/or continual introductions of conspecifics. Such data can inform management interventions in the *ex‐situ* environment and highlight potential contributions of introduced populations to native‐range conservation. We examined multiple metrics of genetic health of a non‐native population of *ca*. 200 critically endangered Yellow‐crested Cockatoos ( *Cacatua sulphurea* )—native to the eastern Indonesian archipelago—introduced to Hong Kong (HK) from the 1960s. We sequenced whole genomes and mitochondrial DNA of HK cockatoos and compared them to native‐range *C. sulphurea* , Sulphur‐crested Cockatoos ( *C. galerita* ) from Australia, and representatives from five additional wild parrot populations. Results indicate HK's cockatoos may have as yet evaded the severe negative genetic consequences of small, isolated populations, and currently have *N*_*e*_ and nucleotide diversity comparable to other wild parrot populations. Their genetic characteristics conform to those of young, mixed‐origin, non‐native populations: high individual‐level variance in relatedness and inbreeding/outbreeding, and influences of admixture. Given that *C. sulphurea* subspecies exhibit very low genetic distances, HK's city cockatoos are worthy of further examination as a potential genetic reservoir and rescue source for *C. sulphurea* .

## 1 Introduction

Global wildlife trade facilitates the introduction of species into new geographic areas across international borders (Levine and D'Antonio 2003; Westphal et al. 2008; Reino et al. 2017). In particular, the live exotic pet trade has introduced diverse taxa to novel environments (Chiron et al. 2009; Lockwood et al. 2019). Cities are frequently the sites of such introductions due to their central position in trade networks.

Negative consequences of species introductions, such as detrimental impacts on native biota (e.g., Strubbe et al. 2009), have traditionally dominated academic discourse on introduced species. However, this overlooks the potentially important function of urban areas as refuges or ‘biodiversity arks’, sustaining species disappearing in their native habitats (Ives et al. 2016; Gibson and Yong 2017; Shaffer 2018) and functioning as reservoirs for increasingly rare genetic lineages. Non‐native city populations may contribute to the conservation of threatened species as stable, safe populations—and as a potential source of candidate individuals for translocations for genetic rescue of native‐range populations, which may be experiencing an ‘extinction vortex’ of population decrease and inbreeding depression (Clarke et al. 2024; Gilpin and Soulé 1986).

However, the conservation value of an introduced city population depends on its long‐term genetic viability, fitness, and structure. For example, small urban populations may suffer the genetic consequences associated with a limited gene pool due to few founding individuals (Barton and Charlesworth 1984; Dussex et al. 2021) or restricted mate choices leading to inbreeding, loss of genetic diversity, and lowered fitness of offspring (Daniell and Murray 1986; Ralls et al. 2018; Kardos et al. 2023). Additionally, populations consisting of individuals originating from breeding facilities may have lowered genetic diversity as an artefact of serial genetic bottlenecks in captivity (Athrey 2018).

It is also possible that trade‐derived, non‐native city populations may contain conspecific individuals of disparate origin, possibly belonging to different subspecies or closely related congeneric species. Successful breeding between these individuals may lead to admixture or genetic introgression in the non‐native population—potentially eroding unique genetic characters or local adaptations of distinct populations (e.g., Leroy et al. 2024). These processes also occur among wild populations of highly mobile species such as birds which are often associated with large and moving ‘hybrid zones’ (Rheindt and Edwards 2011). Human‐mediated transfers related to trade are known to accelerate such introgressive gene flow in birds (e.g., Li et al. 2010). Therefore, when assessing the conservation value of introduced city populations and their potential to function as a source of candidate individuals for reintroduction for genetic rescue, the extent of admixture and its potential impact on the genetic integrity of native‐range populations should be evaluated.

Non‐native populations may also benefit from positive genetic impacts based on the unique manner in which they arise. If the population is derived from unrelated wild‐sourced individuals or is frequently restocked with new individuals from diverse backgrounds, outbred mate pairs can augment genetic diversity through allelic admixture masking recessive deleterious alleles in progeny (Tallmon et al. 2004; Edelaar et al. 2015; Sadanandan et al. 2020; Olah et al. 2021). Although exceptions exist (e.g., Hedrick et al. 2013), long‐term genetic fitness data pre‐ and post‐translocation shows that genetic diversity tends to increase with the introduction of individuals from new backgrounds—whether human‐assisted or natural (Clarke et al. 2024; Miller et al. 2020). For example, re‐distribution of genetic material among Mauritius Parakeet ( *Psittacula echo* ) populations reduced the loss of rare alleles in the remaining small, fragmented populations (Raisin et al. 2012).

One non‐native city population with the potential to benefit conservation is found in central Hong Kong (HK), which currently hosts ~10% of the remaining global population of the critically endangered Yellow‐crested Cockatoo ( *Cacatua sulphurea* ; Gibson and Yong 2017). This city population became established from the 1960s from released or escaped cagebirds (Andersson et al. 2021). Now the population is estimated to have 150–200 individuals (Leven and Corlett 2004), largely residing in the highly urbanised city centre on the north side of Hong Kong island, with therefore limited impact on native flora and fauna (Andersson 2023). The HK cockatoos are designated *C. sulphurea* as opposed to the morphologically similar *C. galerita* from Australia due to their notably smaller body size, which is ~350 g for *C. sulphurea* versus > 700 g for *C. galerita* (Del Hoyo et al. 1992). Hong Kong's *C. sulphurea* population increased considerably 1981–1992, during which time Indonesia exported over 96,700 *C. sulphurea* for the international pet market (Cahill et al. 2006).

Native‐range *C. sulphurea* in Indonesia are thought to now number as few as 1500–2000 birds (IUCN 2025). These can be assigned to up to four evolutionarily significant units (ESUs), with the following subspecies limits, as per Andersson et al. (2025). On Sulawesi, Tukangbesi and the Flores Sea islands, are approximately 300 cockatoos attributed to the subspecies *C. s. sulphurea* (ESU 1; Reuleaux et al. 2022). Up to 1700, treated as *C. s. occidentalis* (ESU 2), persist on the main island chain of the Lesser Sundas ranging from Lombok in the west to Alor in the east—although populations have been extirpated from many islands here (Reuleaux et al. 2022). There are also estimated to be 800–1320 individuals on Sumba island (ESU3; Reuleaux et al. 2022), hosting a taxon of *C. sulphurea* with a distinctive orange crest, *C. s. citrinocristata*, which is considered a separate species by some taxonomic authorities (AviList Core Team 2025), and is therefore not included in the total IUCN population estimate for *C. sulphurea* —despite research demonstrating limited genomic differentiation between orange‐ and yellow‐crested populations (Andersson et al. 2025). Finally, 430–990 individuals are estimated to be surviving on Timor, Rote and Semau islands (Reuleaux et al. 2022), representing a fourth ESU, *C. s. parvula*. A genetic study of *C. sulphurea* museum specimens found that the population of cockatoos on the remote Masalembu island—where contemporary populations are thought to number 17–22 individuals – were likely not to belong to *C. sulphurea* (Andersson et al. 2025), though they are still referred to as *C. s. abbotti* by some taxonomic authorities (AviList Core Team 2025).

To date there has been no research to examine the genetic characteristics of HK's *C. sulphurea* population, despite its potential contributions to the global conservation of the species. The aim of this study was to resolve the two primary questions we note above, that is to (1) understand the genetic viability and fitness of HK's contemporary city cockatoo population, for example inbreeding levels, nucleotide diversity, relatedness and reproductive success, and (2) determine its composition, for example which *C. sulphurea* subspecies are represented therein and in which proportions and/or admixture levels, to indicate their potential contributions to the conservation or genetic recovery of native‐range populations. To this end, we sequenced 41 HK cockatoos and compared them to museum specimens representing the four *C. sulphurea* subspecies in addition to a sample from Masalembu sequenced in a previous study (Andersson et al. 2025). To provide a healthy wild cockatoo population as a baseline to compare population genetic metrics against, we used sequences of an Australian population of Sulphur‐crested Cockatoos (*C. g. galerita*) sequenced in a previous study (Sands et al. 2024), as well as representatives of five additional wild species from the order Psittaciformes, also sequenced in a previous study (Hains et al. 2022). As such we assessed the relative genetic diversity, inbreeding and admixture levels of the HK cockatoo population, and produced data to inform conservation management for dwindling and fragmented native‐range *C. sulphurea* populations.

## 2 Materials and Methods

### 2.1 Sampling and DNA Extraction

**FIGURE 1:**
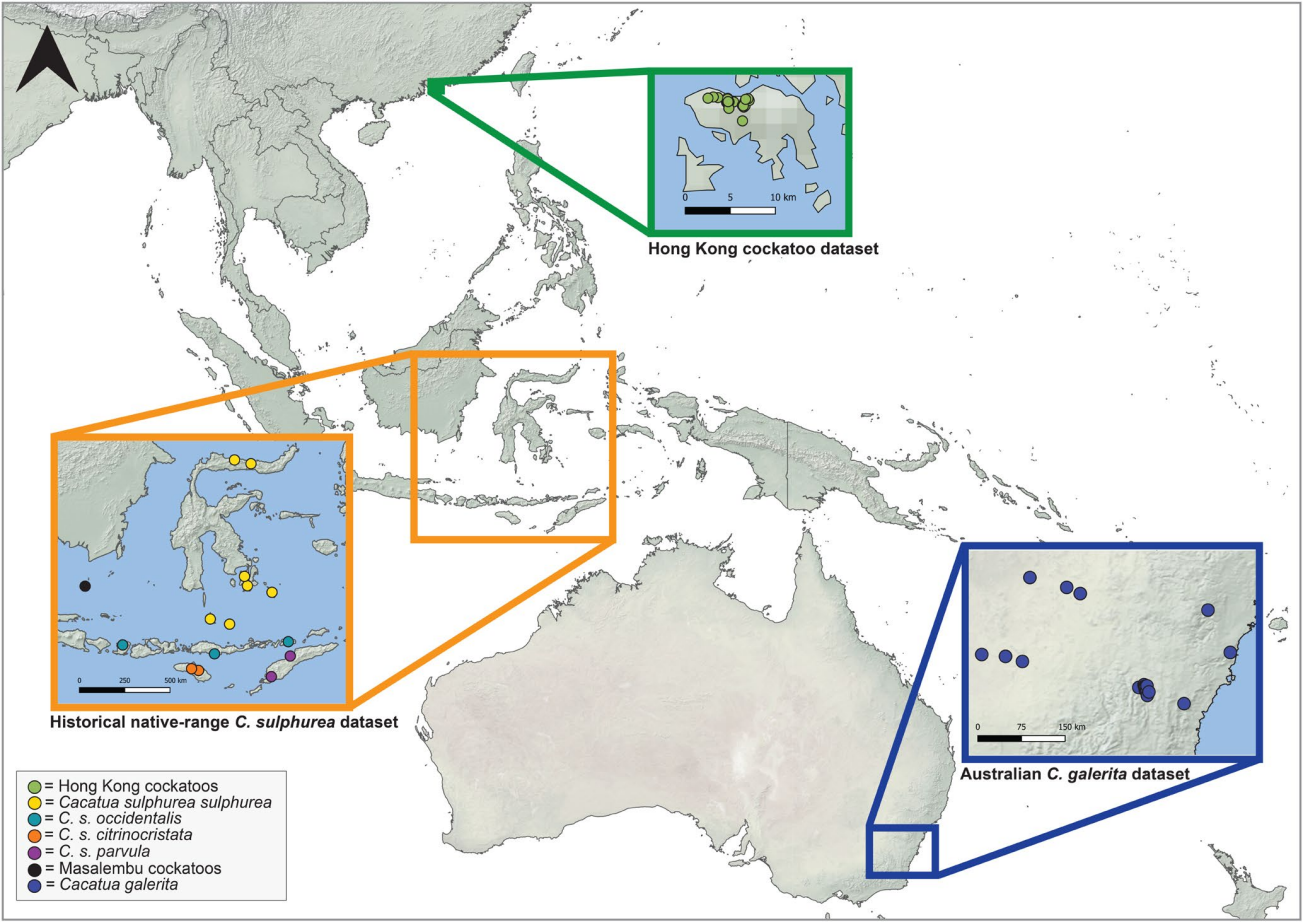
The green inset box shows the source locality of the 41 cockatoo samples included in this study from Hong Kong island, comprising the ‘HK cockatoo dataset’. The orange inset box shows the geographic origins of 15 of the *Cacatua sulphurea* museum samples used in this study coloured by their subspecies as per Andersson et al. (2025), with one additional museum sample incorporated in the study not shown due to unconfirmed locality data. These samples comprise the ‘historical native‐range *C. sulphurea* dataset’. The blue inset box shows the geographic origin of the 28 *C. galerita galerita* genetic samples, a subset from Australian Capital Territory and New South Wales, Australia, from the larger dataset published in Sands et al. (2024). These samples comprise the ‘Australian *C. galerita* dataset’.

From 2018 to 2022, we collected tissue and blood samples from 41 individuals in HK's introduced cockatoo population that were either injured birds taken to rehabilitation centres in HK, trapped and released (permit ref.: (51) AF GR CON 09/51 Pt.7 and (74) AF GR CON 09/51), or died of natural causes. These samples will hereafter be collectively referred to as the ‘HK cockatoo dataset’. The cockatoos are limited to urban parks and fringe forest mainly on the northern and western side of the 78.5 km^2^ HK island (Leven and Corlett 2004; Andersson et al. 2021), with all samples in this study collected within this range (Figure 1).

In addition, we utilised sequences from two previous studies. These include toepad samples from 16 native‐range *C. sulphurea* museum specimens across Indonesia from Andersson et al. (2025) representing all four subspecies of *C. sulphurea* (Figure 1). These samples were included so that it could be determined which island populations the HK cockatoos are likely to have originated from, as well as to determine levels of admixture, if any. We refer to this dataset as the ‘historical native‐range *C. sulphurea* dataset’. We also used 28 museum specimens of *Cacatua galerita galerita* from the Australian Capital Territory and New South Wales, Australia, from Sands et al. (2024). This dataset is hereafter referred to as the ‘Australian *C. galerita* dataset’. For details on DNA extraction and the genomic library protocol for specimens in the historical native‐range *C. sulphurea* dataset, see Andersson et al. (2025), and for the Australian *C. galerita* dataset, see Sands et al. (2024).

DNA extractions for the HK cockatoo dataset were carried out on both tissue and blood samples using a DNeasy Blood and Tissue Kit (QIAGEN) following the manufacturer's recommendations. Quality of the genomic DNA extract was assessed by nanodrop to ensure it met quality and quantity standards for sequencing. Samples were sent to BGI (HK) for 150 bp paired‐end PCR‐free library preparation, with an average insert size of 350 bp, constructed using the MGIEasy PCR‐Free DNA Library Prep Set and sequenced to 10–30× coverage (~11–33Gb) on the DNBSEQ‐G400 platform.

### 2.2 Whole Genome Sequence Processing

The two contemporary sample datasets—HK cockatoos and Australian *C. galerita* —were processed in the same way. First, sequencing adapters were trimmed using AdapterRemoval v2.2.2 (Schubert et al. 2016). Alignment of raw reads was performed with default parameter settings using Burrow Wheelers Aligner (BWA mem; Li 2013), and all were mapped to the annotated reference genome of a *C. sulphurea citrinocristata* individual from Sumba island (NCBI, BioSample accession number: SAMEA114245581), see Supporting Information for assembly method. Merging, sorting, and duplicate marking was done with PicardTools v.2.18.21 (broadinstitute.github.io/picard/). Indexing of BAM files and printing of mapping success statistics was carried out with SAMtools (Li et al. 2009).

To call single nucleotide polymorphisms (SNPs), we used DeepVariant (Poplin et al. 2018), which outperforms other SNP discovery methods in processing time and exhibits a reduced rate of false positives and Mendelian error (Abdelwahab et al. 2023; Behera et al. 2024; Lin et al. 2022). For quality assessment, the report for the Genomic Variant Call Format (GVCF) files for each sample was checked to ensure that the transition‐to‐transversion (Ti/Tv) ratio remained above 1.8 (Lin et al. 2022). Since all samples had a Ti/Tv ratio above 2.3, we proceeded to combine GVCFs to produce a multi‐sample Variant Call Format (VCF) file for each of the two contemporary sample datasets using GLnexus with the ‘DeepVariantWGS’ configuration (Yun et al. 2020). We removed SNPs on the sex chromosomes (W and Z) for subsequent analyses using BCFtools 1.16 (Danecek et al. 2021) ‐‐regions command.

For the HK cockatoo dataset SNPs, we used VCFtools 0.1.16 (Danecek et al. 2021) to remove indels and missing data (‐‐remove‐indels, ‐‐max‐missing 1), to enforce minimum and maximum read depths (‐‐min‐meanDP 4 ‐‐max‐meanDP 23), to ensure SNPs were present in ≥ 2 individuals (‐‐maf 0.0488) and in keeping with Hardy Weinberg equilibrium (‐‐hwe 0.0001). This resulted in a dataset of 2,693,547 SNPs. We then pruned for linkage disequilibrium (LD), which was carried out using a correlation coefficient higher than 0.2 as measured in PLINK 1.90 (Chang et al. 2015), a window size of 50 and a step size of 5 (‐‐indep‐pairwise 50 5 0.2), ultimately retaining 209,704 high‐quality unlinked SNPs for downstream analyses. To filter the Australian *C. galerita* dataset, we used the same parameters but adjusted the ‐‐min‐meanDP to 3, the ‐‐max‐meanDP to 20, and the ‐‐maf to 0.07 to accommodate the smaller sample size. After LD pruning we retained 339,508 SNPs.

To run a principal component analysis (PCA) and generate an Admixture plot, we created a combined dataset comprising the historical native‐range *C. sulphurea* samples with a subset of 20 unrelated HK cockatoo samples to avoid biases introduced by relatedness. This combined dataset will be referred to as the ‘HK cockatoo and historical native‐range *C. sulphurea* dataset’. To avoid batch effects when analysing historical and contemporary samples together, we downsampled all the HK cockatoo BAM files to 3× coverage using PicardTools DownsampleSam. We then re‐processed all samples using a pipeline appropriate for historical and degraded samples and to eliminate damaged and low‐quality genotype information (Andersson et al. 2025). In brief, adapters were removed with AdapterRemoval, FastQ Screen (Wingett and Andrews 2018) was used to screen raw reads for contamination, retaining only reads that mapped uniquely to the reference, mate pairs were repaired with BBTools repair.sh (sourceforge.net/projects/bbmap/), and raw reads were aligned using BWA mem. Merging, sorting, and duplicate marking was done with PicardTools, and indexing of BAM files with SAMtools. We then examined BAM files for *post‐mortem* damage and rescaled them using mapDamage2 (Jónsson et al. 2013).

To call SNPs for the HK cockatoo and historical native‐range *C. sulphurea* dataset, we used genotype likelihood calling in ANGSD (Korneliussen et al. 2014) using the SAMtools genotype model (−GL 1), with parameters set to control for low‐quality data, including: (1) only retaining reads that map uniquely to the reference genome (–uniqueOnly 1), (2) removing reads that are marked as duplicates or that have a low base quality score (−remove_bads 1), (3) removing 15 potentially degraded base pairs on either end of the read (−trim 15), (4) filtering out reads that are not properly paired (−only_proper_pairs 1), (5) only retaining SNPs with a polymorphism *p*‐value less than 1 × 10^–6^ (–SNP_pval 1e‐9) to ensure high‐confidence variant calls, (6) removing reads with a mapping quality score below 20 (−minMapQ 20) and a base quality score below 20 (−minQ 20), (7) requiring a minor allele frequency of 0.01 to remove sequencing errors (−minMaf 0.01), and (8) requiring that a SNP is present in at least 78% of the individuals included. We also set a minimum read depth coverage of 1 for each individual sample (−setMinDepthInd 1) and minimum read depth of 1 required for a genotype call at a given position (across all samples) using −geno_minDepth 2.

We then removed positions on sex chromosomes and 17 unassigned scaffolds using BCFtools view, and used VCFtools to filter the dataset by applying MAF thresholds to ensure that minor alleles were present in ≥ 2 individuals and that SNPs were within Hardy Weinberg equilibrium to 0.0001. We also removed sites with more than 10% missing data (‐‐max‐missing 0.9). We then conducted LD pruning by the same means as mentioned above, after which the SNP file for the HK cockatoo and historical native‐range *C. sulphurea* dataset contained 6024 SNPs.

Raw reads for five wild individuals of species from the order Psittaciformes were downloaded from NCBI, including: Blue‐headed Macaw ( *Primolius couloni* ; GCA_024331605.1), Little Corella ( *Cacatua sanguinea* ; GCA_030265285.1), Maroon‐bellied Parakeet ( *Pyrrhura frontalis* ; GCA_010014865.2), Solomons Cockatoo ( *Cacatua ducorpsii* ; GCA_025448155.1), and Sulphur‐breasted Parakeet (*Aratinga maculata*; GCA_024363045.1), published in Hains et al. (2022). Since they were also derived from museum samples, we processed raw reads for these samples using the same pipeline—but without downsampling, ultimately producing BAM files for these species with coverages ranging from 13.7–21.7× (Tables 1 and S1). For the alignment step, we mapped each species' reads to their corresponding fasta file, also downloaded from NCBI. For the purpose of comparing these Psittaciformes with the HK cockatoo and Australian *C. galerita* cockatoo datasets, we re‐processed all HK cockatoo and Australian *C. galerita* dataset samples > 8.5× through the pipeline for historical and degraded samples described above without the downsampling step. We used BAM files from this pipeline to calculate nucleotide diversity.

**TABLE 1:** Summary information of samples used in this study including data on coverage range and averages, localities, sample sizes, sample sources and collection years.

Locality	Sample size	Population status	Scientific name	Sample source	Coverage range	Coverage $\bar{x}$	Collection timeframe
**Hong Kong**	41	Introduced	*Cacatua sulphurea*	Contemporary population	5.2–19.7	10.8	2018–2022
**Indonesia** (Alor, Buton, Flores, Kalao Toea, Kayuadi, Lombok, Muna, Sulawesi, Sumba, Timor, Tomia)	16	Native	*C. sulphurea*	Museum	1.4–14.8	5.4	1896–1948
**Indonesia** (Masalembu)	1	Undetermined	*C. s. abbotti*	Museum	NA	2.9	1907
**Australia** (Australian Capital Territory, New South Wales)	28	Native	*C. galerita galerita*	Museum	8.8–17.3	9.5	1960–2020
**Suriname**	1	Native	*Aratinga maculata*	NCBI/Museum	NA	18.7	2007
**Solomon Islands**	1	Native	*C. ducorpsii*	NCBI/Museum	NA	21.7	NA
**Australia**	1	Native	*C. sanguinea*	NCBI/Museum	NA	14.5	2002
**Brazil**	1	Native	*Primolius couloni*	NCBI/Museum	NA	16.1	1999
**Paraguay**	1	Native	*Pyrrhura frontalis*	NCBI/Museum	NA	13.7	NA

*Note:* Coverage and SD data apply to nuclear DNA. For more detailed information on individual samples, including collection coordinates, sex, subspecies designation, sample loan/donor institutions, and individual sequence coverage and standard deviations, see Table S1. Abbreviations: NA, not available; NCBI, National Center for Biotechnology Information.

### 2.3 Mitogenomic Analyses

To obtain information about the number of maternal lineages and diversity of mitochondrial haplotypes in the HK cockatoo population, and to see if they match with any haplotypes found in the native range, we generated BAM files for the mitogenomes of each HK cockatoo and native‐range museum sample. We generated BAM files following the same pipeline as outlined above for nuclear DNA on contemporary sample datasets—HK cockatoos and Australian *C. galerita* —mapping to an unpublished reference *C. galerita* mitogenome generated *de novo* using MitoZ (Meng et al. 2019) from a specimen of the Australian National Wildlife Collection, Canberra—see Supporting Information for details. We then created two fasta files per sample using the ANGSD −doFasta 2 −doCounts 1 and −trim 2 commands. Here, one fasta file was generated with lenient quality requirements (−minQ 10) and the other with stringent quality requirements (−minQ 35 −minMapQ 50) for each sample. We used the Clustal Omega 1.2.2 multiple alignment method (Sievers et al. 2011) in Geneious Prime V 2025.2 (geneious.com) to compare the stringent and lenient setting fasta files for each sample. By referring to these side by side we were able to fill in any gaps present in the stringent fasta file sequences with information from the corresponding lenient fasta file sequences, as the latter may be more complete, although with less certainty around calls. After validation, we retained the fasta sequence with stringent settings for each individual. Individual gene fastas for COI, ND2 and CytB were then extracted and exported using Geneious Prime, and haplotype networks were generated using ggplot2 (Gómez‐Rubio 2017) in R V 2024.12 (R Core Team 2021) using a custom script available at github.com/CacSul.

### 2.4 Whole Genome Population‐Genomic Analyses

For the HK cockatoo dataset we generated a relatedness network based on a genomic relationship matrix with dartR (Mijangos et al. 2022), which estimates realised additive genetic relationships. The matrix reports the proportion of alleles shared identical by descent (IBD) between pairs of individuals. The estimates were used to characterise the familial relationships within the HK cockatoo population. The plot was generated using R with a script available at github.com/CacSul.

To calculate contemporary effective breeding population size (*N*_*e*_), we used the programme Current NE (Santiago et al. 2024) on the HK cockatoo dataset SNP VCF, specifying 39 chromosomes.

For inbreeding analyses, we calculated runs of homozygosity (RoH) using Plink 1.90, with the following parameters: ‐‐homozyg‐kb 100 ‐‐homozyg‐window‐snp 25 ‐‐homozyg‐window‐threshold 0.05 ‐‐homozyg‐window‐het 2 ‐‐homozyg‐density 50 ‐‐homozyg‐window‐missing 5 ‐‐homozyg‐het 750. The proportion of the genome carrying RoH (F_ROH_) was calculated by dividing the total length of RoH (KB) for each individual by the total length of the autosomal genome (946,657.613 KB). To test for significant differences in F_ROH_ (and nucleotide diversity) between the two contemporary cockatoo populations we performed non‐parametric tests since the HK dataset was not normally distributed. These were performed in R, and include the unpaired Wilcoxon rank‐sum test (using the wilcox.test function) and Fligner‐Killeen non‐parametric test for homogeneity of variances R function. Finally, to calculate whether inbreeding events happened many or few generations ago, we had to identify appropriate MB length categories for long, medium and short RoH particular to our target species' life‐history traits (Ceballos et al. 2018). To do this, we used the following formula to calculate the coalescent times of RoHs: g = 100/(2rL) (Thompson 2013), where ‘g’ represents the expected time (in generations) back to the common ancestor when the identical‐by‐descent haplotypes forming an RoH coalesce. This estimate can be calculated using the RoH length ‘L’, and the recombination rate ‘r’, which was based on an estimate derived for the Kakapo ( *Strigops habroptilus* ) of 2 cM/Mb (Urban et al. 2024). The generation time used was 14.3 years for *C. sulphurea* (BirdLife International 2021). Using this formula it was determined that an RoH < 1 MB would represent historical inbreeding (estimated at > 375 years ago or 25 generations) and long RoHs > 5 MB represent recent inbreeding < 71 years ago (or 5 generations).

We also generated autosomal PCAs for the HK cockatoo and historical native‐range *C. sulphurea* dataset SNPs. To calculate eigenvectors and eigenvalues we used the Plink 1.90 command ‐‐pca. To generate the 2D and 3D PCA plots we used ggplot2 in R, see github.com/CacSul.

To further explore genetic relationships between the HK cockatoos and their native‐range counterparts, Admixture analyses were conducted to estimate individual admixture proportions among possible groupings (*K*). NGSadmix (Skotte et al. 2013) was implemented on the HK cockatoo and historical native‐range *C. sulphurea* dataset genotype likelihood file for a set number of *K* ranging from 1 to 9. To compute the optimum *K*, we followed the method detailed in ngsAdmix_tutorial.md available at github.com/alexkrohn/AmargosaVoleTutorials. In brief, we ran NGSadmix 10 times per *K* value (*K =* 1 through *K =* 9), created a file of all the log likelihoods and formatted it for CLUMPAK (Cluster Markov Packager Across K; Kopelman et al. 2015). The file was then imported into CLUMPAK to estimate the best *K*, using methods developed by Evanno et al. (2005). Using R, scenarios *K =* 2–5 were plotted as the most supported and realistic among assessments.

Finally, we calculated nucleotide diversity for the HK cockatoo and Australian *C. galerita* datasets. We first used ANGSD to produce a 1D site frequency spectrum (SFS) file for both datasets. For this, we provided a contig map and used the –doSaf 1 and realSFS ANGSD functions with the following filters: –uniqueOnly 1 –remove_bads 1 –minMapQ 30 –minQ 30 –trim 15 –only_proper_pairs 1 –setMinDepth 2. This SFS output was run through an R script available at github.com/Nopaoli/Demographic‐Modelling/tree/master/Diversity_fromSFS to calculate nucleotide diversities. With the same method, we also calculated nucleotide diversities for each of the museum‐derived *C. sulphurea* subspecies, the NCBI parrot species, as well as for each individual in the HK cockatoo and Australian datasets. We only included samples > 8.5× coverage to avoid bias introduced by low‐coverage samples (Kardos and Waples 2025).

## 3 Results

### 3.1 Relatedness

**FIGURE 2:**
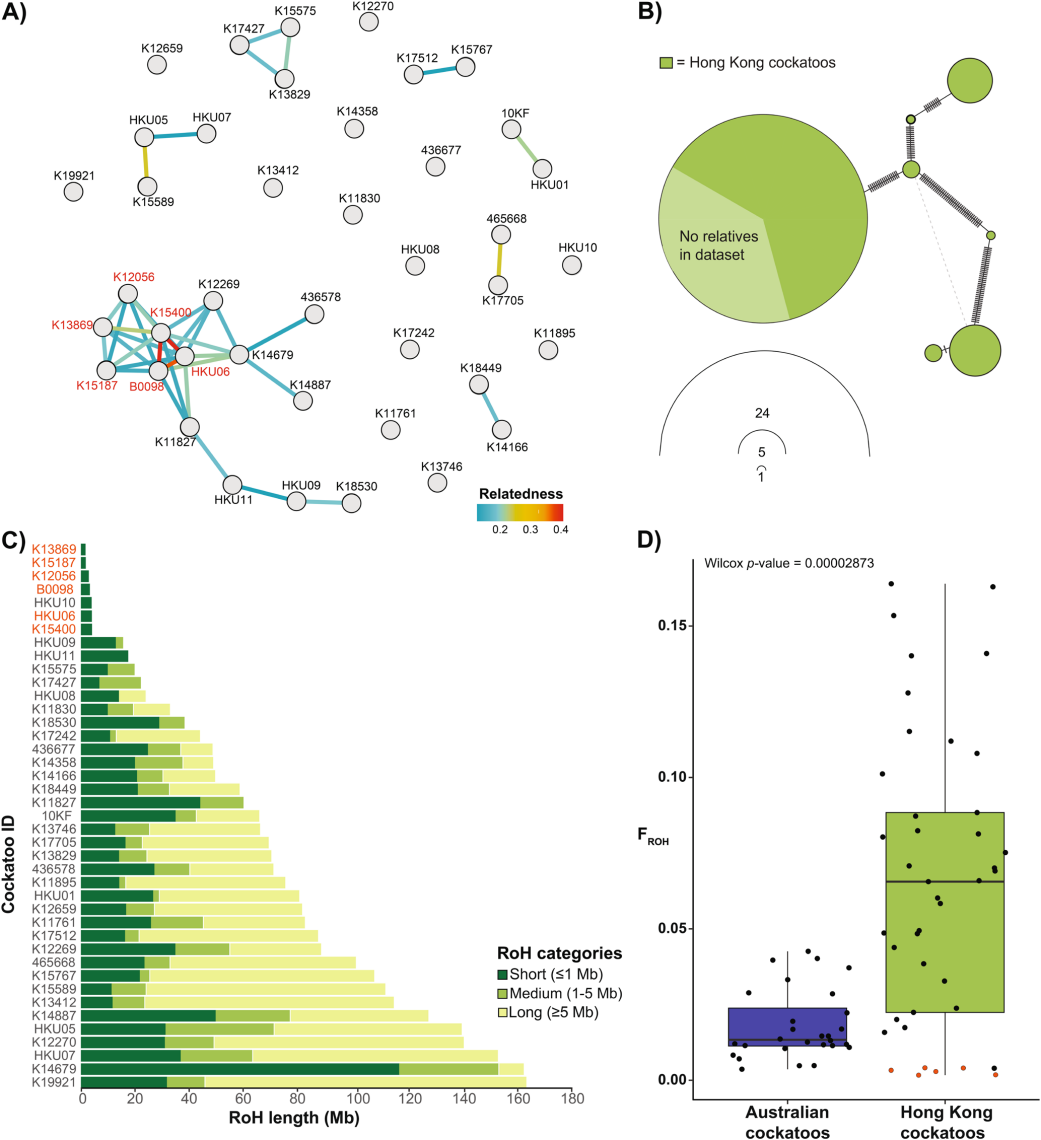
(A) Relationship network based on a genomic relationship matrix generated with dartR V2 in R from 209,704 single nucleotide polymorphisms (SNPs) by estimating the pairwise covariance of genotypes, and the probability that alleles at a random locus are identical by descent. Each individual Hong Kong (HK) cockatoo is represented by a circle and labelled with sample ID. Individual IDs labelled in red correspond with individuals in Figure 2C labelled in red. (B) Haplotype network for mitochondrial gene CytB (1142 base pairs) for the 41 HK cockatoos, generated in R. Light green segment indicates the proportion of the 24 individuals in the large haplotype group that do not have any relatives in the dataset. (C) Summed lengths of short (≤ 1 Mb), medium (1–5 Mb), and long (≥ 5 Mb) runs of homozygosity (RoH) per individual cockatoo from the 41 sample HK population dataset. Samples are ordered by decreasing total RoH from top to bottom. (D) F_ROH_ is the proportion of the genome that contains RoH, dots in red correspond to the cockatoo individuals in red in Figure 2A,C. Boxplots show the average F^ROH^ and sample dispersion for cockatoos in Hong Kong and Australia. Result of non‐parametric unpaired Wilcoxon rank‐sum test indicated in top left of plot.

The dartR IBD analysis (Figure 2A) shows that 28 out of 41 individuals (68.3%) in the HK cockatoo dataset are related to some degree, and that three individuals (K15400; HKU06; B0098) have the highest degree of relatedness. This analysis also reveals a large family group, involving 14 of the 41 samples, and six other trios or pairwise relatives, whilst 13 out of 41 individuals (32%) have no relatives. The mitochondrial DNA (mtDNA) haplotype network for CytB shows seven unique haplotypes in our dataset of 41 HK cockatoos, with one haplotype shared by 24 individuals (Figure 2B). Of the group of 24 cockatoos that share the same CytB haplotype, nine are unrelated to any other cockatoos in the HK cockatoo dataset. For other mtDNA gene haplotype networks for the HK population, see Figure S1.

### 3.2 Inbreeding

Average F_ROH_ in the HK cockatoo population is 0.065, whilst for our Australian *C. galerita* dataset it is 0.018. The distribution of F_ROH_ values in the HK cockatoo dataset is significantly broader than in the Australian cockatoos (Fligner‐Killeen:med chi‐squared = 21.92, df = 1, *p <* 0.001), and the mean F_ROH_ is significantly higher in the HK population (Wilcoxon two‐sample test, W = 243, df = 1, *p <* 0.001; Figure 2D). In the HK cockatoo dataset, seven individuals have almost no RoH (indicated in red in Figure 2C) and correspond with the highly related familial cluster from Figure 2A. Other individuals have a relatively high F_ROH_; some of these have many long fragments of RoH (e.g., individual K19921), indicating inbreeding in the past five generations, or 71 years, since the HK cockatoo population became established. Yet others, meanwhile, have more short fragments (e.g., K14679), indicative of inbreeding many generations ago, since long RoHs have been repeatedly broken down over generations by recombination events (Ceballos et al. 2018). Very short or non‐existent RoH can also indicate parents with dissimilar genetic sequences (such as parents from different subspecies) resulting in less identical regions of the maternally and paternally inherited chromosome copies.

### 3.3 Effective Population Size

The current effective population size for HK cockatoos was estimated to be *N*_*e*_ = 27.88 (90% CI: 24.52–31.69). Given that the census size (*N*_*C*_) of the entire Hong Kong population is estimated to be ~200 individuals (Andersson et al. 2021), this suggests an *N*_*e*_ */N*_*C*_ ratio of ~14%. Current NE also identified 12 relationships in the dataset (Table S2), all of which correspond with relationships identified by dartR analysis (Figure 2A).

### 3.4 Mitogenomic Analyses

**FIGURE 3:**
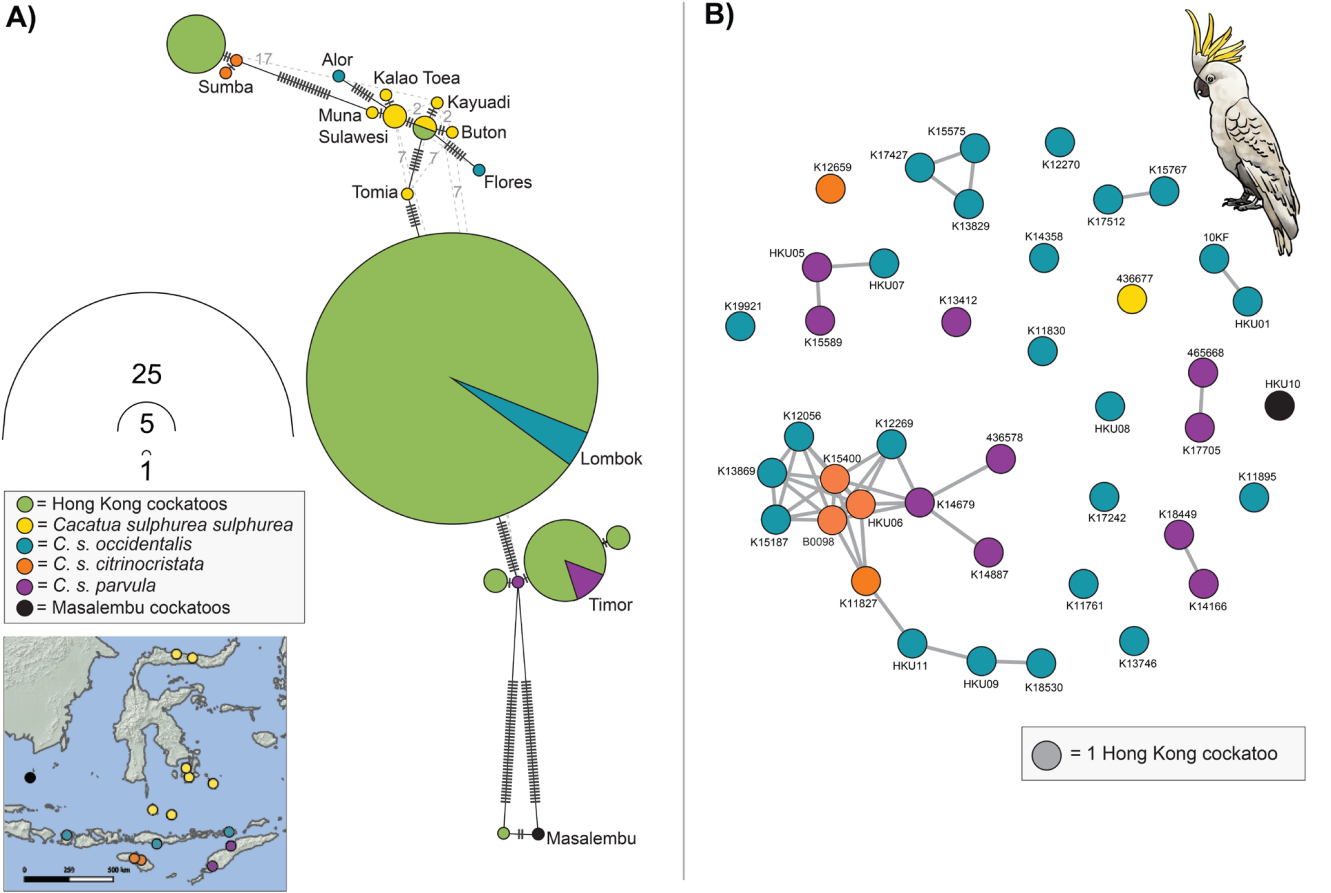
(A) Haplotype network for mitochondrial DNA (mtDNA) gene CytB for museum specimens of 16 *Cacatua sulphurea* , one cockatoo from Masalembu island, and 41 Hong Kong cockatoos. The *C. sulphurea* samples are coloured according to which subspecies they belong to as per Andersson et al. (2025) and are shown on the inset map and labelled with the source island. See Figure S1 for additional mtDNA genes ND2 and COI. (B) Genomic relationship matrix network generated with dartR V2 in R for 41 individual Hong Kong cockatoo samples, coloured according to the *C. sulphurea* subspecies their CytB haplotype aligns with.

When we combined the CytB gene haplotypes for the HK cockatoo dataset with the *C. sulphurea* museum samples, we found that the HK population shares haplotypes with museum samples representing all four *C. sulphurea* subspecies and the Masalembu island cockatoo. This indicates that the maternal lineage of individuals in the HK population can be traced back to specific islands in the native range—such as Lombok, Timor, and Sulawesi (Figure 3A). The closely related family group contains individuals with haplotypes from three different *C. sulphurea* subspecies (Figure 3B).

### 3.5 Population Structure

**FIGURE 4:**
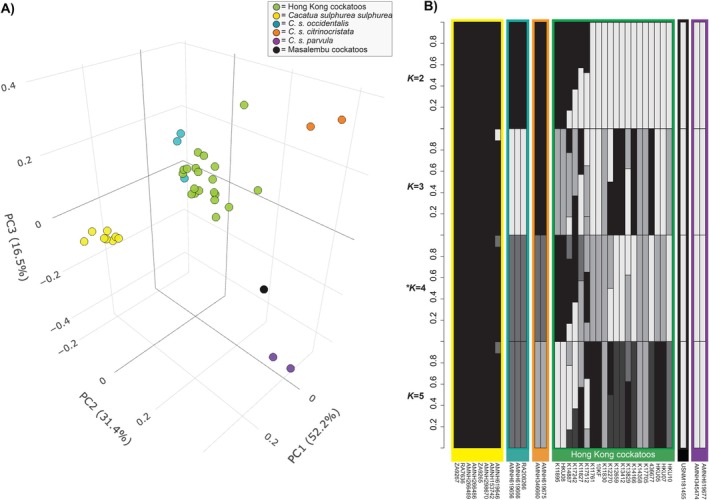
(A) A 3D space principal component analysis (PCA) plot generated from 6024 single nucleotide polymorphisms (SNPs) showing the first three components including 16 native‐range *Cacatua sulphurea* specimens and 20 unrelated individuals from the introduced Hong Kong population. Samples are coloured according to subspecies affinities as indicated in the legend and described in Andersson et al. (2025). See Figure S2 for a 2D PCA. (B) Admixture plot showing values of *K* from 2 to 5 calculated with NGSadmix using the same SNP dataset; the most‐favoured *K* value (i.e., *K* = 4) is indicated with an asterisk. The subspecies groupings are indicated on the X axis and with boxes coloured in accordance with the legend in panel 4A.

The PCA analysis based on genome‐wide SNPs using the HK cockatoo and historical native‐range *C. sulphurea* dataset revealed that the 20 unrelated HK cockatoos included in this analysis are concentrated in the genomic space in between all the different *C. sulphurea* subspecies—with many individuals appearing to be most closely affiliated with *C. s. occidentalis*, encompassing populations from the main island chain of the Lesser Sundas ranging from Lombok in the west to Alor in the east (Figure 4A). This corresponds with patterns observed in the haplotype network for the mtDNA gene CytB (Figure 3A), as well as COI and ND2 (Figure S1), which demonstrates that the dominant haplotype in the HK population originates from the Lesser Sunda Island chain. In the Admixture analyses (Figure 4B), all *K* scenarios reflect the HK cockatoos are of mixed origin with genetic contributions from the various *C. sulphurea* subspecies, in keeping with results from the RoH (Figure 2C,D) and mtDNA (Figure 3B) analyses, which both indicate admixture. For a PCA which also includes three *C. g. galerita* samples, see Figure S3, which shows no indications of introgression with *C. g. galerita* in the HK cockatoo dataset.

### 3.6 Nucleotide Diversity

**FIGURE 5:**
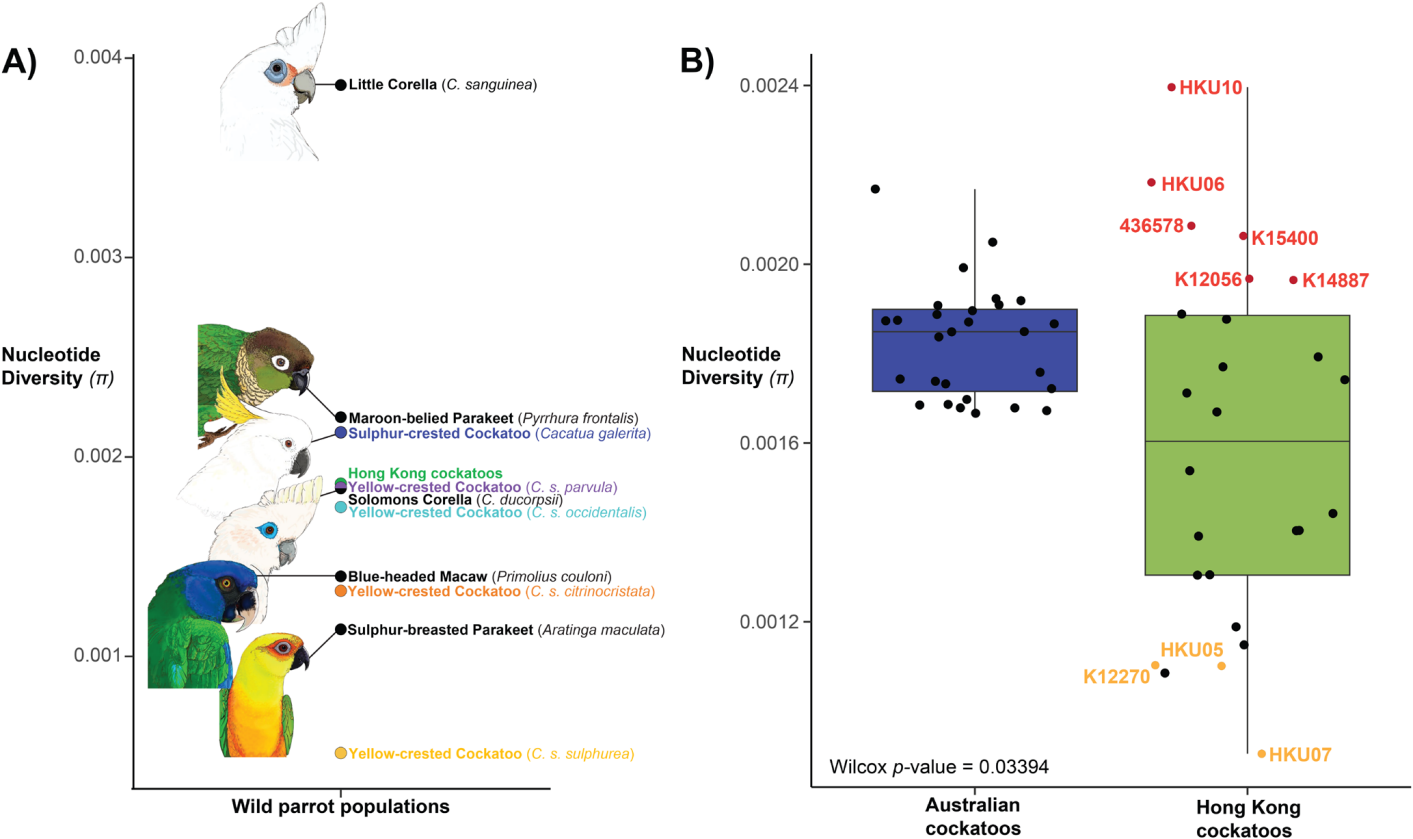
(A) A comparison of nucleotide diversity among wild populations of Psittaciformes sampled in this and other studies (Sands et al. 2024; Andersson et al. 2025; Hains et al. 2022), and also sourced from NCBI. Art by Patricia J. Latas. (B) Boxplot showing average and dispersal of individual‐level nucleotide diversity in the cockatoo datasets from Hong Kong and Australia, calculated by the same means. The coloured dots with adjacent sample ID names correspond to individuals with the least (red) and most (yellow) runs of homozygosity, or inbreeding, as shown in Figure 2C. For both plots A and B, all samples used were > 8.5× coverage, processed in a pipeline designed for low‐coverage museum‐derived data, and nucleotide diversity was calculated using ANGSD SFS.

We estimated nucleotide diversity for the HK and Australian cockatoo datasets both overall and on an individual level (Figure 5B), and compared them to other wild parrot populations (Figure 5A). The human‐introduced HK population only had slightly lower average nucleotide diversity (0.0018) than the wild population of *C. g. galerita* from Australia (0.0021) used here as a reference for a natural cockatoo population. Overall nucleotide diversity for the contemporary HK cockatoo dataset was also not dissimilar to the representatives of wild populations of *C. s. occidentalis*, *C. s. citrinocristata* and *C. s. parvula*. Averages of nucleotide diversity are significantly different between the HK and Australian populations (Wilcoxon two‐sample test, W = 487, df = 1, *p = 0.0339*), and there is significantly more variation in nucleotide diversity among individuals in the HK cockatoo dataset (Fligner‐Killeen:med chi‐squared = 22.783, df = 1, *p <* 0.001) (Figure 5B) – with admixed individuals (shown in red on the plot) having the highest nucleotide diversity and inbred individuals with the highest F_ROH_ (shown in yellow) having the lowest nucleotide diversity. Broad distribution in nucleotide diversity is expected in small, introduced, mixed‐origin populations, whereas natural populations would be more consistent as seen in the Australian cockatoo dataset.

## 4 Discussion

With Earth's biota currently undergoing accelerated, human‐induced species loss (Ceballos et al. 2015), novel, bold, and unconventional actions must be considered to aid species survival (Lundgren et al. 2024). Some have considered assessing the suitability of hitherto unexplored resources to aid conservation, such as remnant ‘biodiversity arks’ (Meng et al. 2021), including those of introduced species in anthropogenic environments (Shaffer 2018).

By obtaining genetic data from HK's resident non‐native population of critically endangered *C. sulphurea*, we assessed (1) the overall genetic viability, fitness and structure of the HK cockatoo population and (2) what this information indicates about the conservation value of this population for its native‐range counterparts. Our results suggest that currently, the *N*_*e*_ and nucleotide diversity in the introduced HK cockatoo population are comparable to in situ wild populations of congener species, while specific individuals show evidence of admixture/inbreeding (Figures 2 and 5). The data demonstrate that the HK population presents what may be common genetic characteristics of young, mixed‐origin, introduced populations: high individual‐level variance in relatedness and inbreeding, as well as high levels of admixture between *C. sulphurea* subspecies (e.g., Kolbe et al. 2008). Since previous research on *C. sulphurea* has shown that subspecies are separated by shallow nuclear sequence divergence of < 0.148% (Andersson et al. 2025), this suggests that HK's population is genetically viable, and could still be useful to conservation interventions such as reintroduction, despite admixture—particularly those individuals that exhibit low levels of inbreeding and high nucleotide diversity.

For a population to be genetically viable, a critical mass or portion must be effectively breeding in unrelated mate pairs to avoid the deleterious genetic effects of inbreeding. The *N*_*e*_ of the HK population (27.88, or between 24.52–31.69, individuals) is above the 10% *N*_*e*_ suggested to be sufficient to sustain diversity (Franklin 1980)—but it should be noted that *N*_*e*_ estimates below 50 indicate an unstable population (Hoban et al. 2020), and it is estimated that optimally an *N*_*e*_ of between 500 to 1000 is required for populations to retain evolutionary potential (Franklin and Frankham 1998; Frankham et al. 2014). This suggests there would still be cause for concern for the future of the HK cockatoo population, as well as many other populations of endangered species that do not meet this threshold. Meanwhile, the HK cockatoos' average F_ROH_—indicative of inbreeding prevalence—is significantly higher than the baseline value for *C. g. galerita* from Australia (Figure 2D) and other wild bird populations. For example, wild Lesser White‐fronted Geese ( *Anser erythropus* ) sampled in Russia and Norway have an F_ROH_ < 0.02 (Díez‐del‐Molino et al. 2020) while average F_ROH_ in HK's non‐native urban cockatoos (< 0.07) is more akin to captive‐origin Lesser White‐fronted Geese in Sweden (Díez‐del‐Molino et al. 2020), but lower than the F_ROH_ of translocated populations of Kakapo in New Zealand (Dussex et al. 2021). However, it should be noted that there is a wide variation in F_ROH_ among HK individuals, with some having almost zero F_ROH_ (Figure 4A), bringing down the overall average. Nucleotide diversity in the HK cockatoo dataset is also similar to other wild psittacine populations (Figure 5A). Overall this suggests that HK's population of cockatoos has not yet lost significant genetic diversity due to genetic drift or inbreeding.

Seven CytB mtDNA haplotypes were identified in the HK cockatoo dataset, with some rare lineages represented therein. Haplotypes from all four subspecies of native‐range *C. sulphurea* are present (Figure 3A,B), and one HK individual's mtDNA aligns most closely with the cockatoo from Masalembu island—where there is a contemporary population of fewer than 22 individuals. Haplotype networks of COI and ND2 also demonstrate the same patterns (Figure S1). Over half the HK cockatoo dataset (24 individuals) carry a CytB haplotype identical to the *C. sulphurea* museum specimen from Lombok island, where cockatoo populations are now extirpated. Lombok was one of the initial target islands for *C. sulphurea* trapping and trade (Reuleaux et al. 2022). Of the 24 HK cockatoos with the Lombok haplotype, nine are unrelated. This suggests that multiple cockatoos from Lombok (or potentially also neighbouring islands such as Sumbawa that are unsampled in our dataset) have been introduced to HK, some breeding successfully and others not.

The influence of admixture between different *C. sulphurea* subspecies is evident in the HK cockatoos included in this study. The diversity of *C. sulphurea* mtDNA haplotypes in the HK population, even within a highly related familial group (Figure 3B), is a strong indication of admixture between *C. sulphurea* subspecies. This pattern is echoed in the genome‐wide analyses, where some individuals have long segments of RoH, indicating inbreeding within the last 71 years (or five generations), but others have little‐to‐no RoH, suggesting they are progeny of parents from different populations. Although the subset of 20 unrelated HK cockatoos is most genetically akin to *C. s. occidentalis*—which encompasses cockatoo populations on the string of islands from Lombok to Alor (Figure 1)—they primarily occupy space in‐between the various *C. sulphurea* subspecies (Figures 4A, S2, and S3), suggesting that the HK population contains offspring produced by parents that originate from different islands. The admixture analysis reflects the same, with the HK individuals sharing genetic material from multiple subspecies under all *K* scenarios (Figure 4B). As such, since its introduction in the 1960s, the HK population has provided an approximate 60‐year (> 4 generations) test case for the genetic implications of gene flow among *C. sulphurea* subspecies. Interestingly, the cluster of highly related individuals in the HK cockatoo dataset is also the most likely to be admixed, carrying haplotypes from multiple subspecies and exhibiting the lowest levels of F_ROH_ and highest levels of nucleotide diversity—potentially suggesting that admixture may have translated to breeding success, although further studies are required to confirm this.

The continual introduction of individuals from disparate native‐range islands over several decades may have added new genetic material to the HK population, facilitating admixture and elevating nucleotide diversity in this otherwise small and isolated cockatoo population. Within‐population genetic diversity tends to increase with the number of source populations (Kolbe et al. 2008), as is reflected in the fact that despite its small size, the HK cockatoo population has a similar nucleotide diversity to multiple other wild parrot populations—both on an average level, and for multiple individuals (Figure 5A,B). Admixed individuals have elevated nucleotide diversity (Figure 5B), suggesting they may have a greater potential to adapt to changing environments, as a broader range of gene variants are available. However, admixture‐related benefits to genetic fitness may only be evident in the first three generations (Frankham 2016), after which a delayed onset of outbreeding depression may become apparent in subsequent generations (Kovach et al. 2015). It should be noted that overall F_ROH_ in the HK cockatoo dataset is relatively high, and some individuals do have long segments of RoH indicating recent inbreeding, with genetic consequences that may only manifest in progeny in future generations (e.g., Adams et al. 2011). There is already some evidence of this process, since three individuals which have among the highest F_ROH_ in the HK population also have lower nucleotide diversity than the other individuals (Figure 5B). Therefore, continuous monitoring of the HK population is crucial when assessing its genetic viability and potential contribution to native‐range conservation (e.g., Kelly et al. 2021).

Admixture in the HK cockatoo population will have caused a degree of ‘genetic contamination’ between *C. sulphurea* subspecies, arguably undesirable for the conservation of unique lineages. However, given that some populations and even subspecies of *C. sulphurea* may be close to extinction, an introduction of *ex‐situ* individuals could be one of the only viable options for their survival. For example, the near‐extinct nominate subspecies *C. s. sulphurea* has lost 77% of its population since 1950, is down to just a few hundred individuals fragmented in small populations across multiple islands, and has been suggested to be likely go extinct without intervention (Reuleaux et al. 2022). There is a high degree of nuclear‐genomic similarity among *C. sulphurea* subspecies, with sequence divergences of < 0.148% (Andersson et al. 2025) – well below the 0.5% ‘grey zone’ of speciation delineated by Roux et al. (2016) – which makes the species more suitable for genetic rescue via candidates from multiple, or mixed, subspecies origin.

*Cacatua sulphurea* adheres to all but one of the guidelines for success in genetic rescue, which recommends gene flow among populations that are: conspecific, similarly adapted, with no chromosomal differences, and optimally only isolated from each other by around 500 years (Frankham et al. 2011; Frankham 2015). Though subspecies of *C. sulphurea* have been separated by > 500 years, there is evidence of successful genetic rescue using different subspecies diverged by > 50,000 years (Pavlova et al. 2023). The positive genetic fitness metrics presented here for some individuals in the contemporary HK cockatoo population suggest that this population can make future contributions to native‐range *C. sulphurea* conservation. However, enhanced monitoring would be required to exclude inbred individuals, and further long‐term research would be necessary on any genetically fit admixed individuals to rule out negative genetic consequences associated with interbreeding among subspecies. Our results provide important baseline data for authorities weighing whether the benefits of introducing admixed *C. sulphurea* to the native range would outweigh the consequences of the alternative scenario of inaction—which would likely be extinction for many *C. sulphurea* populations or even subspecies. At any rate, a multitude of conservation challenges faced by *C. sulphurea* would need to be addressed before considering genetic rescue, including mitigation of threats in their native range, biosecurity, and the logistical and financial realities of such conservation interventions. Additionally, there may be divergence in behaviour and ecology that may impact the suitability of individuals from Hong Kong's cockatoo population for conservation purposes. As such, genetic work conducted in this study merely provides foundational data on the possibility of such endeavours.

Practitioners who work with other parrot and bird species facing similarly dire conservation scenarios have considered interventions that utilise or produce mixed‐origin populations. For example, *ex‐situ* testing for the impact of genetic rescue between Helmeted Honeyeaters (*Lichenostomus melanops cassidix*) and Yellow‐tufted Honeyeaters (*L. m. gippslandicus*) was initiated in 2017 in an effort to evade Helmeted Honeyeater extinction within 50 years (Pavlova et al. 2023). These different subspecies are now being interbred, despite being ecologically, morphologically and behaviourally distinct, with an estimated divergence time of 56,000 years—an action which has ultimately increased genetic fitness in terms of breeding rates, nestlings raised, and chick sex ratios, and which has produced negligible evidence of outbreeding depression (Pavlova et al. 2023). Additionally, interspecific hybridisation with a congener species has been proposed as a genetic intervention for the near‐extinct Orange‐bellied Parrot ( *Neophema chrysogaster* ), numbering only 81 individuals in a population breeding in Tasmania (Silver et al. 2025; Hogg et al. 2021). This is seen by practitioners as one of the only strategies available to alleviate the extreme bottleneck and loss of genetic diversity in the Orange‐bellied Parrot, and to reverse the 87% chance of the species becoming extinct in 20 years (Woinarski et al. 2024).

Long‐term data monitoring beyond the F3 generation in the context of successful human‐manufactured cross‐species or ‐subspecies genetic rescue programmes in mammals, reptiles or birds remains rare (Frankham 2015), but some examples exist. For instance, genetic rescue interventions in the Florida Panther ( *Puma concolor coryi* ) prevented its extirpation through the release of eight female Pumas (*P. c. stanleyana*) from Texas in 1995 (Johnson et al. 2010), which after three decades of observation, or five generations, has increased the abundance of the rescued population more than fivefold, and *N*_*e*_ > 20×, whilst still maintaining allele frequencies that distinguish Florida Panthers from all other North American Pumas (Onorato et al. 2024).

However, as mentioned, it would be important to carefully consider specific candidate individuals for translocation and genetic rescue. Though there is evidence that introducing individuals from smaller, even inbred, source populations still has the effect of reversing the deleterious genetic impact of bottlenecks, for example in South Island Robins ( *Petroica australis* ; Heber et al. 2013) and Kakapos (Dussex et al. 2021), it is nevertheless important to exercise caution when selecting individuals for genetic rescue, as the introduction of new genetic material to a population may not always produce the expected results of augmenting diversity long term, and is dependent on the genetic characteristics and number of individuals that are translocated, for example as in Isle Royale Wolves ( *Canis lupus* ; Robinson et al. 2019). Therefore future research on the HK cockatoo population should focus on other factors important in determining suitability for native‐range introduction, for example the frequency, nature and impact of deleterious mutations, disease prevalence, and the age of individuals.

## 5 Conclusions

The information presented in this study provides an overview of the genetic fitness and structure of HK's introduced cockatoo population, determining that this population should not be dismissed as ecologically redundant, and is worthy of further examination for its potential contributions to global *C. sulphurea* conservation. Not only do HK cockatoos provide a function as a wild *C. sulphurea* ‘back up’ population, they also showcase a natural four‐generation experiment of the genetic consequences of interbreeding *C. sulphurea* subspecies in the wild. However, both introduced populations and the alternative *ex‐situ* approach of captive breeding have important limitations to consider—for example, though captive breeding allows for the possibility of more close monitoring and control over mate‐pair selection, in some cases there may be limited founding individuals and behavioural or even genetic adaptation to captivity in the potential candidates produced (Frankham et al. 2002). Wild populations, meanwhile, are not always possible to manage and there may be behavioural adaptations of urban birds that prevent successful translocation. Further studies are required to fully determine whether gene flow among *C. sulphurea* subspecies is genetically favourable before concluding definitively that HK's largely admixed cockatoo population can indeed have a positive impact to native‐range *C. sulphurea* conservation in terms of providing candidate individuals for reintroduction and genetic rescue in Indonesia. If such action is considered, then it should be accompanied by a thorough risk‐assessment process and management framework for systematic long‐term monitoring.

## Funding

This work was supported by funding from the University of Hong Kong (J.M., C.D.), HKU Robert Whyte Research Fund (J.M., A.F.S.), and Research Grants Council of Hong Kong, grant number 17104824 (J.M., A.F.S.), Faculty of Science, HKU (J.M.) and Wildlife Reserves Singapore (C.D., A.A.A.). Some of the work at NUS was funded by a Singapore National Research Foundation (NRF) grant to F.E.R. (NRF‐NRFI07‐2021‐0008).

## Conflicts of Interest

The authors declare no conflicts of interest.

## Supporting information


**Table S1:** Samples used in the study with information on their collection locality, sex and sequence coverage. Samples from Kansas University Natural History Museum (KU), Yale Peabody Museum (YPM), Louisiana Museum of Natural Science (LSUMZ), Field Museum of Natural History (FMNH) were downloaded from NCBI. Subspecies names and designations for *Cacatua sulphurea* follow Andersson et al. (2025).
**Table S2:** Relationships in the HK cockatoo dataset identified by CurrentNe and dartR analyses. First degree denotes either parent–offspring or full sibling relationship and corresponds to a proportion of alleles that are identical by descent (IBD) of ~0.4 or above. Second degree relationships include grandparent and grandchild, half‐siblings, or uncle/aunt and nephew/niece, and correspond to an IBD value of ~0.25 to 0.38. Third degree relationships are first cousins, and have an IBD of 0.125 to 0.24.
**Figure S1:** Haplotype network for mitochondrial DNA (mtDNA) genes CytB, ND2 and CO1 for museum specimens of 16 *Cacatua sulphurea* , 1 cockatoo from Masalembu island, and 41 Hong Kong cockatoos. An additional concatenated haplotype network with all 3 mtDNA genes is provided, with source islands labelled. The *C. sulphurea* samples are coloured according to which subspecies they belong to as per Andersson et al. (2025) and are shown on the inset map.
**Figure S2:** A 2D space principal component analysis (PCA) plot showing the first two components and including 16 native‐range *Cacatua sulphurea* specimens and 20 unrelated individuals from the introduced HK cockatoo population, generated from 6024 single nucleotide polymorphisms (SNPs). Samples coloured according to subspecies affinities as indicated in the legend and in Andersson et al. (2025).
**Figure S3:** A 2D space principal component analysis (PCA) plot showing the first two components and including 16 native‐range *Cacatua sulphurea* specimens and 20 unrelated individuals from the introduced HK cockatoo population, as well as three individuals from sister species *C. g. galerita*, generated from 83,872 single nucleotide polymorphisms (SNPs). Samples are coloured according to subspecies as indicated in the legend and described in Andersson et al. (2025).

## Data Availability

Scripts relevant to this paper can be found at github.com/AstridAlexAndersson/CacSul and data can be accessed on Figshare DOI 10.6084/m9.figshare.30646658.
