# Supplementary material for: Assessing the Genetic Health and Conservation Value of an Introduced Urban Population of a Critically Endangered Parrot
Source: Evol Appl. 2026 May 14;19(5):e70245. doi: 10.1111/eva.70245 (PMC13176652; doi:10.1111/eva.70245)
Supplement: Supplementary file 1 — Table S1: Samples used in the study with information on their collection locality, sex and sequence coverage. Samples from Kansas University Natural History Museum (KU), Yale Peabody Museum (YPM), Louisiana Museum of Natural Science (LSUMZ), Field Museum of Natural History (FMNH) were downloaded from NCBI. Subspecies names and designations for Cacatua sulphurea follow Andersson et al. (2025). Table S2: Relationships in the HK cockatoo dataset identified by CurrentNe and dartR analyses. First degree denotes either parent–offspring or full sibling relationship and corresponds to a proportion of alleles that are identical by descent (IBD) of ~0.4 or above. Second degree relationships include grandparent and grandchild, half‐siblings, or uncle/aunt and nephew/niece, and correspond to an IBD value of ~0.25 to 0.38. Third degree relationships are first cousins, and have an IBD of 0.125 to 0.24. Figure S1: Haplotype network for mitochondrial DNA (mtDNA) genes CytB, ND2 and CO1 for museum specimens of 16 Cacatua sulphurea , 1 cockatoo from Masalembu island, and 41 Hong Kong cockatoos. An additional concatenated haplotype network with all 3 mtDNA genes is provided, with source islands labelled. The C. sulphurea samples are coloured according to which subspecies they belong to as per Andersson et al. (2025) and are shown on the inset map. Figure S2: A 2D space principal component analysis (PCA) plot showing the first two components and including 16 native‐range Cacatua sulphurea specimens and 20 unrelated individuals from the introduced HK cockatoo population, generated from 6024 single nucleotide polymorphisms (SNPs). Samples coloured according to subspecies affinities as indicated in the legend and in Andersson et al. (2025). Figure S3: A 2D space principal component analysis (PCA) plot showing the first two components and including 16 native‐range Cacatua sulphurea specimens and 20 unrelated individuals from the introduced HK cockatoo population, as well as three individua [file EVA-19-e70245-s001.docx]

**Assessing the genetic health and conservation value of an introduced urban population of a critically endangered parrot**

**SUPPLEMENTARY MATERIALS**

**Reference genome – BioSample Accession number: SAMEA114245581**

Reads were mapped to a chromosome-level reference genome for the Citron-crested Cockatoo (*Cacatua sulphurea citrinocristata*) assembled by Oxford Nanopore long-read sequencing. High-molecular-weight DNA was extracted from a blood sample from a captive female, and sequenced on PromethION flow cell. Omni-C chromatin capture libraries were then generated for Hi-C–based scaffolding.

Bases for the raw Nanopore reads were called with Guppy 6.5.7 (nanoporetech.com/software/other/guppy), filtered for quality and *de novo* assembled with Flye 2.9.5 (Kolmogorov et al. 2019). The assembly was then refined and improved using Medaka 2.0.1 (github.com/nanoporetech/medaka) and Racon 1.5.0 (Vaser et al. 2017). To identify and remove haplotypic duplications Purge_dups v1.2.6 (Guan et al. 2020) was used. Next the Hi-C reads were aligned to the assembly via BWA 0.7.18 (Li and Durbin 2009), and chromosome-scale scaffolding was conducted using YaHS 1.2.2 (Zhou et al. 2023). The assembly was then manually curated following rapid curation guidelines (gitlab.com/wtsi-grit/rapid-curation). We then ran BUSCO 5.8.0 (Manni et al. 2021) with the aves_odb10 dataset which assessed 99.2% complete BUSCOs. There were 41 chromosomes (39 autosomes, Z and W), the mitochondrial genome, and 90 unassigned scaffolds.

**Mitochondrial reference genome construction**

The mitochondrial genome came from raw DNBseq paired-end reads (BGI, Hong Kong) from specimen B34682 (*Cacatua galerita*; Australian National Wildlife Collection) which were randomly subsampled down to 20% using Seqtk 1.3-r106 (github.com/lh3/seqtk). It was then *de novo* assembled (plus read filtering, mitoscaffold identification, gene annotation) using MitoZ 3.5 (Meng et al. 2019) with “all” module selected and default parameters. The final mitogenome had 16,756 base pairs and all 13 annotated protein coding genes.

**Table S1**. Samples used in the study with information on their collection locality, sex and sequence coverage. Samples from Kansas University Natural History Museum (KU), Yale Peabody Museum (YPM), Louisiana Museum of Natural Science (LSUMZ), Field Museum of Natural History (FMNH) were downloaded from NCBI. Subspecies names and designations for *Cacatua sulphurea* follow Andersson et al. (2024).

| **Specimen ID** | **Source** | **Scientific name** | **Subspecies designation** | **Sex** | **Material** | **Latitude (approx)** | **Longitude (approx)** | **Collection locality (source)** | **Cov x̄** | **SD** | **Collection date** |
| --- | --- | --- | --- | --- | --- | --- | --- | --- | --- | --- | --- |
| **HK COCKATOOS** | | | | | | | | | | | |
| 436578 | SPCA HK | *Cacatua sulphurea* | *NA* | F | muscle | 22.2771 | 114°16'403 | Admiralty, HK | 10.7 | 9.5 | 2018 |
| 436677 | SPCA HK | *Cacatua sulphurea* | *NA* | F | muscle | 22.2771 | 114°16'403 | Admiralty, HK | 17.1 | 25.0 | 2018 |
| 465668 | SPCA HK | *Cacatua sulphurea* | *NA* | F | muscle | 22.2771 | 114°16'403 | Admiralty, HK | 10.5 | 31.7 | 2019 |
| 10KF | KFBG | *Cacatua sulphurea* | *NA* | M | blood | 22.2771 | 114°16'403 | Admiralty, HK | 10.7 | 9.5 | 2021 |
| B0098 | KFBG | *Cacatua sulphurea* | *NA* |  | blood | 22.2771 | 114°16'403 | Admiralty, HK | 9.0 | 12.9 | 2019 |
| HKU01 | HKU | *Cacatua sulphurea* | *NA* | M | blood | 22.2771 | 114°16'403 | Admiralty, HK | 10.6 | 11.9 | 2020 |
| HKU05 | HKU | *Cacatua sulphurea* | *NA* | M | blood | 22.2771 | 114°16'403 | Admiralty, HK | 10.2 | 9.8 | 2021 |
| HKU06 | HKU | *Cacatua sulphurea* | *NA* | F | blood | 22.2771 | 114°16'403 | Admiralty, HK | 10.6 | 10.3 | 2021 |
| HKU07 | HKU | *Cacatua sulphurea* | *NA* | M | blood | 22.2771 | 114°16'403 | Admiralty, HK | 11.0 | 9.9 | 2021 |
| HKU08 | HKU | *Cacatua sulphurea* | *NA* | M | blood | 22.2771 | 114°16'403 | Admiralty, HK | 10.8 | 10.9 | 2022 |
| HKU09 | HKU | *Cacatua sulphurea* | *NA* | F | blood | 22.2771 | 114°16'403 | Admiralty, HK | 10.4 | 11.4 | 2021 |
| HKU10 | HKU | *Cacatua sulphurea* | *NA* | F | blood | 22.2771 | 114°16'403 | Admiralty, HK | 10.2 | 13.5 | 2021 |
| HKU11 | HKU | *Cacatua sulphurea* | *NA* | M | blood | 22.2771 | 114°16'403 | Admiralty, HK | 10.6 | 10.3 | 2020 |
| K11761 | KFBG | *Cacatua sulphurea* | *NA* | M | muscle | 22.2472 | 114°18'11 | Shouson Hill, HK | 8.2 | 8.2 | 2018 |
| K11827 | KFBG | *Cacatua sulphurea* | *NA* | M | muscle | 22.2771 | 114°16'403 | Admiralty, HK | 11.0 | 9.3 | 2018 |
| K11830 | KFBG | *Cacatua sulphurea* | *NA* | M | blood | 22.2876 | 114°14'17 | Sai Ying Pun, HK | 10.9 | 9.6 | 2018 |
| K11895 | KFBG | *Cacatua sulphurea* | *NA* | M | blood | 22.2860 | 114°19'15 | Causeway Bay, HK | 10.9 | 10.4 | 2018 |
| K12056 | KFBG | *Cacatua sulphurea* | *NA* | F | muscle | 22.2684 | 114°18'65 | Happy Valley, HK | 10.2 | 59.9 | 2018 |
| K12269 | KFBG | *Cacatua sulphurea* | *NA* | F | muscle | 22.2771 | 114°16'403 | Admiralty, HK | 7.2 | 14.4 | 2018 |
| K12270 | KFBG | *Cacatua sulphurea* | *NA* | F | muscle | 22.2860 | 114°19'15 | Causeway Bay, HK | 10.0 | 27.9 | 2018 |
| K12659 | KFBG | *Cacatua sulphurea* | *NA* | F | blood | 22.2822 | 114°12'93 | Kennedy Town, HK | 10.6 | 11.0 | 2019 |
| K13412 | KFBG | *Cacatua sulphurea* | *NA* | F | muscle | 22.2860 | 114°19'15 | Causeway Bay, HK | 5.2 | 7.5 | 2019 |
| K13746 | KFBG | *Cacatua sulphurea* | *NA* | F | blood | 22.2860 | 114°19'15 | Causeway Bay, HK | 10.7 | 12.5 | 2019 |
| K13829 | KFBG | *Cacatua sulphurea* | *NA* | M | blood | 22.2860 | 114°19'15 | Causeway Bay, HK | 10.9 | 10.8 | 2019 |
| K13869 | KFBG | *Cacatua sulphurea* | *NA* | M | blood | 22.2771 | 114°16'403 | Admiralty, HK | 10.9 | 10.0 | 2019 |
| K14166 | KFBG | *Cacatua sulphurea* | *NA* | F | blood | 22.2684 | 114°18'65 | Happy Valley, HK | 10.6 | 11.1 | 2019 |
| K14358 | KFBG | *Cacatua sulphurea* | *NA* | M | blood | 22.2678 | 114°23'61 | Chai Wan, HK | 10.9 | 10.2 | 2020 |
| K14679 | KFBG | *Cacatua sulphurea* | *NA* | F | blood | 22.2860 | 114°19'15 | Causeway Bay, HK | 10.6 | 11.9 | 2020 |
| K14887 | KFBG | *Cacatua sulphurea* | *NA* | M | blood | 22.2876 | 114°14'17 | Sai Ying Pun, HK | 21.2 | 18.1 | 2020 |
| K15187 | KFBG | *Cacatua sulphurea* | *NA* | F | blood | 22.2684 | 114°18'65 | Happy Valley, HK | 10.5 | 11.0 | 2020 |
| K15400 | KFBG | *Cacatua sulphurea* | *NA* | M | muscle | 22.2684 | 114°18'65 | Happy Valley, HK | 10.1 | 47.7 | 2020 |
| K15575 | KFBG | *Cacatua sulphurea* | *NA* | F | muscle | 22.2771 | 114°16'403 | Admiralty, HK | 9.9 | 20.5 | 2020 |
| K15589 | KFBG | *Cacatua sulphurea* | *NA* | M | muscle | 22.2860 | 114°19'15 | Causeway Bay, HK | 10.6 | 10.0 | 2020 |
| K15767 | KFBG | *Cacatua sulphurea* | *NA* | M | blood | 22.2771 | 114°16'403 | Admiralty, HK | 11.0 | 10.4 | 2021 |
| K17242 | KFBG | *Cacatua sulphurea* | *NA* | F | muscle | 22.2771 | 114°16'403 | Admiralty, HK | 9.7 | 32.6 | 2021 |
| K17427 | KFBG | *Cacatua sulphurea* | *NA* | F | muscle | 22.2771 | 114°16'403 | Admiralty, HK | 9.8 | 27.1 | 2021 |
| K17512 | KFBG | *Cacatua sulphurea* | *NA* | M | muscle | 22.2771 | 114°16'403 | Admiralty, HK | 19.7 | 27.4 | 2021 |
| K17705 | KFBG | *Cacatua sulphurea* | *NA* | M | muscle | 22.2771 | 114°16'403 | Admiralty, HK | 10.1 | 20.8 | 2021 |
| K18449 | KFBG | *Cacatua sulphurea* | *NA* | F | muscle | 22.2860 | 114°19'15 | Causeway Bay, HK | 10.2 | 34.7 | 2021 |
| K18530 | KFBG | *Cacatua sulphurea* | *NA* | M | blood | 22.2771 | 114°16'403 | Admiralty, HK | 10.6 | 11.4 | 2021 |
| K19921 | KFBG | *Cacatua sulphurea* | *NA* | F | blood | 22.2771 | 114°16'403 | Admiralty, HK | 10.4 | 10.2 | 2022 |
| **HISTORICAL NATIVE RANGE *C. sulphurea*** | | | | | | | | | | | |
| 181455 | USNM | *Cacatua sulphurea* | *NA* | M | toepad | -5.45239 | 114°42'723 | Masalembu , Indonesia | 2.9 | 6.8 | 1907 |
| 619675 | AMNH | *Cacatua sulphurea* | *C. s. citrinocristata* | M | toepad | -9.63469 | 119°84'318 | Sumba, Indonesia | 14.8 | 16.9 | NA |
| 346660 | AMNH | *Cacatua sulphurea* | *C. s. citrinocristata* | F | toepad | -9.72716 | 120°20'172 | Sumba, Indonesia | 5.9 | 13.4 | 1932 |
| 266486 | AMNH | *Cacatua sulphurea* | *C. s. sulphurea* | M | toepad | -6.806588 | 120°47'42.8 | Kayuadi, Indonesia | 2.1 | 6.4 | 1927 |
| RA7636 | RMNH | *Cacatua sulphurea* | *C. s. sulphurea* | F | toepad | NA | NA | No source information | 2.9 | 5.6 | NA |
| 266489 | AMNH | *Cacatua sulphurea* | *C. s. sulphurea* | F | toepad | -7.36896 | 121°78'13 | Kalao Toea, Indonesia | 2.0 | 6.7 | 1927 |
| 619666 | AMNH | *Cacatua sulphurea* | *C. s. occidentalis* | M | toepad | -8.87965 | 121°01'25 | Flores, Indonesia | 9.1 | 12.0 | 1896 |
| 619656 | AMNH | *Cacatua sulphurea* | *C. s. occidentalis* | F | toepad | -8.26357 | 124°76'725 | Alor, Indonesia | 2.7 | 10.5 | 1897 |
| 208266 | RMNH | *Cacatua sulphurea* | *C. s. occidentalis* | F | toepad | -8.3583 | 116°54'17 | Lombok, Indonesia | 1.4 | 2.9 | 1909 |
| 345474 | AMNH | *Cacatua sulphurea* | *C. s. parvula* | M | toepad | -10.04036 | 123°90'409 | Timor, Indonesia | 13.8 | 14.9 | 1932 |
| 619677 | AMNH | *Cacatua sulphurea* | *C. s. parvula* | F | toepad | -8.9916 | 124°86'8 | Timor, Indonesia | 2.2 | 7.2 | 1897 |
| 619646 | AMNH | *Cacatua sulphurea* | *C. s. sulphurea* | M | toepad | -5.75435 | 123°93'189 | Tomia, Indonesia | 10.6 | 12.9 | 1901 |
| 153742 | AMNH | *Cacatua sulphurea* | *C. s. sulphurea* | M | toepad | 0.78742 | 122°86'955 | Sulawesi; Indonesia | 2.2 | 9.3 | 1914 |
| ZA9267 | RMNH | *Cacatua sulphurea* | *C. s. sulphurea* | M | toepad | -5.175 | 122°84'17 | Butung, Indonesia | 2.6 | 5.3 | 1948 |
| ZA9265 | RMNH | *Cacatua sulphurea* | *C. s. sulphurea* | F | toepad | -4.9917 | 122°65'83 | Muna, Indonesia | 2.4 | 4.3 | 1948 |
| 298670 | AMNH | *Cacatua sulphurea* | *C. s. sulphurea* | F | toepad | 0.5851 | 122°1'49 | Sulawesi; Indonesia | 8.3 | 11.1 | 1930 |
| **AUSTRALIAN *C. galerita*** | | | | | | | | | | | |
| B29233 | ANWC | *Cacatua galerita* | *C. g. galerita* | F | liver | -34.5528 | 150°75'78 | New South Wales, Australia | 26.7 | 48 | 2001 |
| B29234 | ANWC | *Cacatua galerita* | *C. g. galerita* | M | liver | -34.5528 | 150°75'78 | New South Wales, Australia | 10.3 | 25.5 | 2001 |
| B34065 | ANWC | *Cacatua galerita* | *C. g. galerita* | F | liver | -33.7167 | 150°31'67 | New South Wales, Australia | 8.8 | 20.1 | 1960 |
| B34139 | ANWC | *Cacatua galerita* | *C. g. galerita* | M | liver | -35.2778 | 149°10'83 | Canberra, Australia | 8.8 | 20.1 | 1960 |
| B34631 | ANWC | *Cacatua galerita* | *C. g. galerita* | M | liver | -35.3333 | 149°15 | Canberra, Australia | 8.8 | 20.1 | 2006 |
| B34682 | ANWC | *Cacatua galerita* | *C. g. galerita* | M | liver | -34.7307 | 146°65'03 | New South Wales, Australia | 8.8 | 20.1 | 2006 |
| B52820 | ANWC | *Cacatua galerita* | *C. g. galerita* | - | liver | -35.19 | 149°05'32 | Canberra, Australia | 8.8 | 20.1 | 2011 |
| B53100 | ANWC | *Cacatua galerita* | *C. g. galerita* | - | muscle | -35.5638 | 149°84'44 | New South Wales, Australia | 8.8 | 20.1 | 2009 |
| B53291 | ANWC | *Cacatua galerita* | *C. g. galerita* | F | liver | -35.2124 | 149°11'17 | Canberra, Australia | 8.8 | 20.1 | 2012 |
| B53583 | ANWC | *Cacatua galerita* | *C. g. galerita* | M | muscle | -35.316 | 149°108 | Canberra, Australia | 8.8 | 20.1 | 2013 |
| B53585 | ANWC | *Cacatua galerita* | *C. g. galerita* | M | muscle | -35.3007 | 149°08 | Canberra, Australia | 8.8 | 20.1 | 2013 |
| B53586 | ANWC | *Cacatua galerita* | *C. g. galerita* | F | muscle | -35.312 | 149°10'88 | Canberra, Australia | 8.8 | 20.1 | 2013 |
| B53846 | ANWC | *Cacatua galerita* | *C. g. galerita* | F | liver | -35.2444 | 148°95 | Canberra, Australia | 8.8 | 20.1 | 1979 |
| B53982 | ANWC | *Cacatua galerita* | *C. g. galerita* | F | liver | -35.40189 | 149°11'794 | Canberra, Australia | 8.8 | 20.1 | 2017 |
| B54825 | ANWC | *Cacatua galerita* | *C. g. galerita* | F | liver | -33.3903 | 147°79'33 | New South Wales, Australia | 8.8 | 20.1 | 2010 |
| B54826 | ANWC | *Cacatua galerita* | *C. g. galerita* | F | liver | -33.3903 | 147°79'33 | New South Wales, Australia | 8.8 | 20.1 | 2010 |
| B54827 | ANWC | *Cacatua galerita* | *C. g. galerita* | F | liver | -33.3903 | 147°79'33 | New South Wales, Australia | 8.8 | 20.1 | 2010 |
| B54828 | ANWC | *Cacatua galerita* | *C. g. galerita* | F | liver | -33.3903 | 147°79'33 | New South Wales, Australia | 8.8 | 20.1 | 2010 |
| B54829 | ANWC | *Cacatua galerita* | *C. g. galerita* | M | liver | -33.2686 | 147°52'53 | New South Wales, Australia | 10.0 | 18.9 | 2010 |
| B54875 | ANWC | *Cacatua galerita* | *C. g. galerita* | F | liver | -33.0741 | 146°79'79 | New South Wales, Australia | 17.3 | 33.1 | 2010 |
| B54897 | ANWC | *Cacatua galerita* | *C. g. galerita* | M | liver | -34.5937 | 145°84'65 | New South Wales, Australia | 9.0 | 26.6 | 2010 |
| B54898 | ANWC | *Cacatua galerita* | *C. g. galerita* | F | liver | -34.6305 | 146°31'57 | New South Wales, Australia | 9.6 | 20.6 | 2010 |
| B54899 | ANWC | *Cacatua galerita* | *C. g. galerita* | F | liver | -34.6305 | 146°31'57 | New South Wales, Australia | 9.5 | 22.8 | 2010 |
| B54900 | ANWC | *Cacatua galerita* | *C. g. galerita* | M | liver | -34.6305 | 146°31'57 | New South Wales, Australia | 10.1 | 21.3 | 2010 |
| B59003 | ANWC | *Cacatua galerita* | *C. g. galerita* | M | liver | -35.2422 | 149°06'73 | Canberra, Australia | 9.7 | 20.4 | 2013 |
| B59123 | ANWC | *Cacatua galerita* | *C. g. galerita* | F | liver | -35.2384 | 149°08'24 | Canberra, Australia | 10.4 | 14.4 | 2018 |
| B59459 | ANWC | *Cacatua galerita* | *C. g. galerita* | - | liver | -35.2237 | 149°06'2 | Canberra, Australia | 10.0 | 16.5 | 2019 |
| B59739 | ANWC | *Cacatua galerita* | *C. g. galerita* | F | heart | -35.2557 | 149°07 | Canberra, Australia | 9.9 | 16.2 | 2020 |
| **NCBI PARROT GENOMES** | | | | | | | | | | | |
| 137433 | YPM | *Aratinga maculata* | NA | M | toe pad | -55.9617 | 2°00'50 | Sipaliwini, Suriname | 18.7 | 62.3 | 2007 |
| 131250 | KU | *Cacatua ducorpsii* | NA | F | tissue | -7.03500 | 155°85'800 | Solomon Islands | 21.7 | 103 | NA |
| 92461 | KU | *Cacatua sanguinea* | *C. s. westralensis* | - | tissue | -24.1200 | 115°26'66 | Western Australia, Australia | 14.5 | 83.7 | 2002 |
| 395540 | FMNH | *Primolius couloni* | NA | M | toe pad | 8.93333 | -72°78'33 | Acre, Brazil | 16.1 | 34.1 | 1999 |
| 25884 | LSUMZ | *Pyrrhura frontalis* | NA | - | tissue | 26.1828 | 56°37'12 | Caazapá, Paraguay | 13.7 | 61.4 | NA |

*Note:* Coverage and SD data apply to nuclear DNA. Abbreviations: Cov, coverage; F, female; M, male; SD, standard deviation; NA, not available, Society for the Prevention of Cruelty to Animals HK (SPCA HK), the University of HK (HKU), Kadoorie Farm and Botanic Gardens (KFBG), the American Museum of Natural History (AMNH); Naturalis Biodiversity Center (RMNH); Smithsonian National Museum of Natural History (USNM); Australian National Wildlife Collection (ANWC), Yale Peabody Museum (YPM), Kansas University Natural History Museum (KU), Field Museum of Natural History (FMNH), Louisiana Museum of Natural Science (LSUMZ).

**Table S2.** Relationships in the HK cockatoo dataset identified by CurrentNe and dartR analyses. First degree denotes either parent–offspring or full sibling relationship and corresponds to a proportion of alleles that are identical by descent (IBD) of ~0.4 or above. Second degree relationships include grandparent and grandchild, half-siblings, or uncle/aunt and nephew/niece, and correspond to an IBD value of ~0.25 to 0.38. Third degree relationships are first cousins, and have an IBD of 0.125 to 0.24.

| **ID 1** | **ID 2** | **Classification of relationship**  **from darT IBD** | **Relationship identified**  **by Current NE** |
| --- | --- | --- | --- |
| B0098 | K15400 | First degree | ✔ |
| HKU06 | K15400 | First degree | ✔ |
| B0098 | HKU06 | First degree | ✔ |
| HKU05 | K15589 | First degree |  |
| 465668 | K17705 | First degree |  |
| K13869 | K15400 | First degree |  |
| 10KF | HKU01 | First degree | ✔ |
| HKU06 | K11827 | First degree |  |
| B0098 | K14679 | First/second degree |  |
| K15187 | K15400 | First/second degree |  |
| HKU06 | K14679 | First/second degree |  |
| K12056 | K15400 | First/second degree |  |
| K12056 | K13869 | Second degree | ✔ |
| K13869 | K15187 | Second degree | ✔ |
| K14679 | K15400 | Second degree |  |
| HKU09 | K18530 | Second degree | ✔ |
| K13829 | K15575 | Second degree | ✔ |
| K13829 | K17427 | Second degree | ✔ |
| K14166 | K18449 | Second degree |  |
| HKU11 | K11827 | Second degree | ✔ |
| K15575 | K17427 | Second degree | ✔ |
| K12269 | B0098 | Second degree |  |
| K14679 | K14887 | Second degree |  |
| K12269 | K15400 | Second degree |  |
| B0098 | K13869 | Second degree |  |
| K12269 | K14679 | Second degree |  |
| HKU06 | K13869 | Second degree |  |
| K12269 | HKU06 | Second degree |  |
| B0098 | K15187 | Second degree |  |
| HKU06 | K12056 | Second degree |  |
| K11827 | K15400 | Second degree |  |
| B0098 | K12056 | Second degree |  |
| HKU06 | K15187 | Second degree |  |
| B0098 | K11827 | Second degree |  |
| K12056 | K15187 | Second/third degree |  |
| 436578 | K14679 | Second/third degree |  |
| HKU09 | HKU11 | Second/third degree | ✔ |
| K15767 | K17512 | Second/third degree |  |
| HKU05 | HKU07 | Second/third degree |  |

**Figure S1.** Haplotype network for mitochondrial DNA (mtDNA) genes CytB, ND2 and CO1 for museum specimens of 16 *Cacatua sulphurea*, 1 cockatoo from Masalembu island, and 41 Hong Kong cockatoos. An additional concatenated haplotype network with all 3 mtDNA genes is provided, with source islands labelled. The *C. sulphurea* samples are coloured according to which subspecies they belong to as per Andersson et al. (2024) and are shown on the inset map.


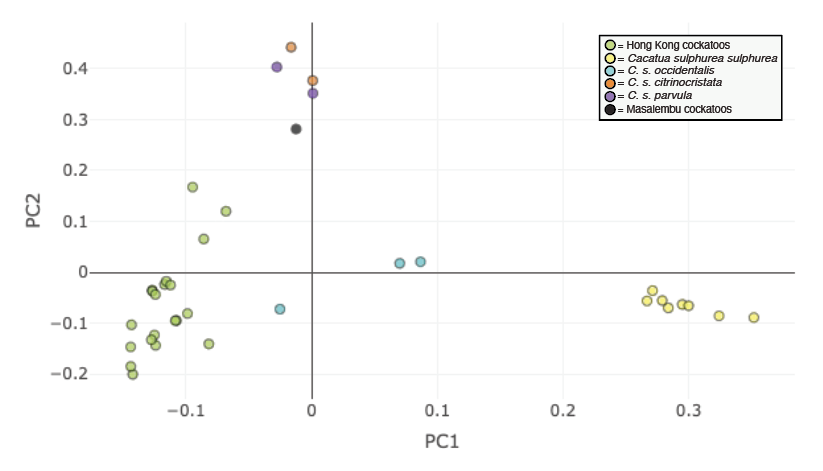


**Figure S2.** A 2D space principal component analysis (PCA) plot showing the first two components and including 16 native-range *Cacatua sulphurea* specimens and 20 unrelated individuals from the introduced HK cockatoo population, generated from 6,024 single nucleotide polymorphisms (SNPs). Samples coloured according to subspecies affinities as indicated in the legend and in Andersson et al. (2024).


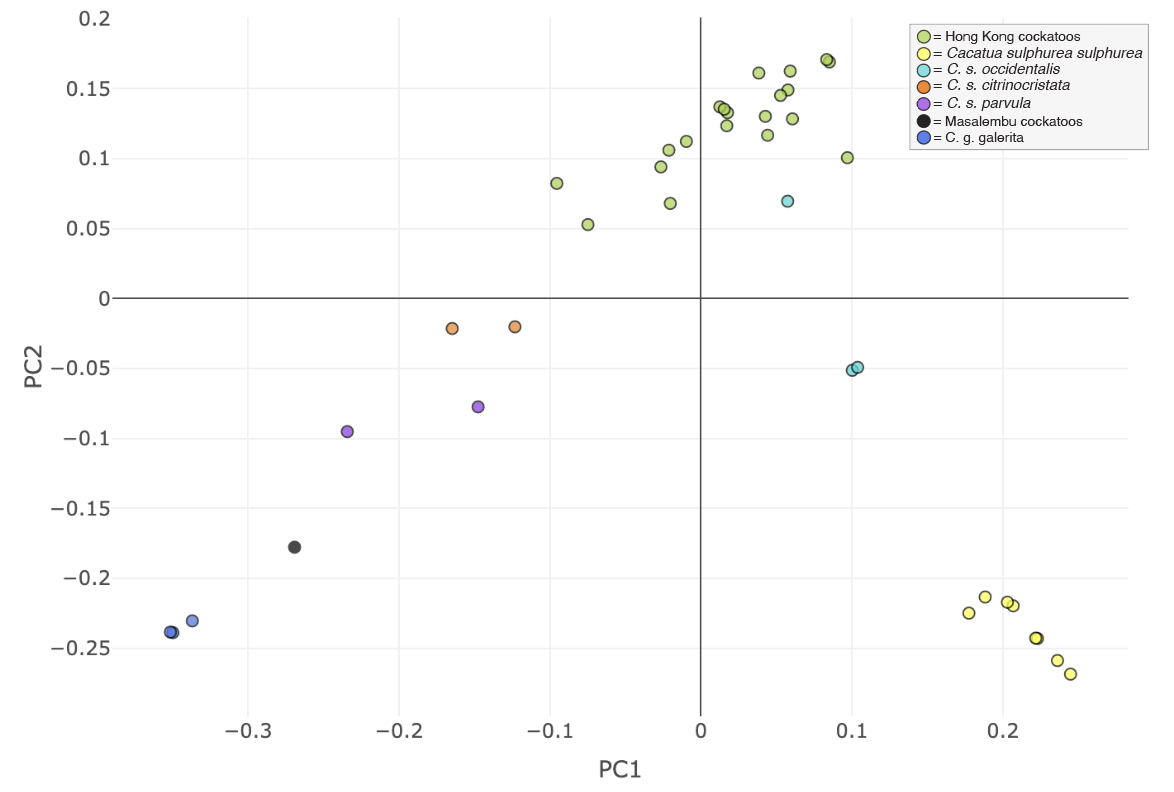


**Figure S3.** A 2D space principal component analysis (PCA) plot showing the first two components and including 16 native-range *Cacatua sulphurea* specimens and 20 unrelated individuals from the introduced HK cockatoo population, as well as three individuals from sister species *C. g. galerita*, generated from 83,872 single nucleotide polymorphisms (SNPs). Samples are coloured according to subspecies as indicated in the legend and described in Andersson et al. (2024).

**References**

Guan D, McCarthy SA, Wood J, Howe K, Wang Y, Durbin R. 2020. Identifying and removing haplotypic duplication in primary genome assemblies. *Bioinformatics*. 36(9):2896–2898.

Li H, Durbin R. 2009. Fast and accurate short read alignment with Burrows-Wheeler transform. *Bioinformatics*. 25(14):1754–1760.

Manni M, Berkeley MR, Seppey M, Simão FA, Zdobnov EM., 2021. BUSCO update: Novel and streamlined workflows along with broader and deeper phylogenetic coverage for scoring of eukaryotic, prokaryotic, and viral genomes. *Mol. Biol. Evol.* 38(10):4647–4654.

Meng G, Li Y, Yang C, Liu S. 2019. MitoZ: A toolkit for animal mitochondrial genome assembly, annotation and visualization. *Nucleic Acids Res.* 47(11):e63.

Kolmogorov M, Yuan J, Lin Y, Pevzner PA. 2019. Assembly of long, error-prone reads using repeat graphs. *Nat. Biotechnol.* 37(5):540–546.

Vaser R, Sovic I, Nagarajan N, Sikic M. 2017. Fast and accurate *de novo* genome assembly from long uncorrected reads. *Genome Res.* 27(5):737–746.

Zhou C, McCarthy SA, Durbin R. 2023. YaHS: Yet another Hi-C scaffolding tool. *Bioinformatics*. 39(1):btac808.
